# Fusion of Wearable Kinetic and Kinematic Sensors to Estimate Triceps Surae Work during Outdoor Locomotion on Slopes

**DOI:** 10.3390/s22041589

**Published:** 2022-02-18

**Authors:** Sara E. Harper, Dylan G. Schmitz, Peter G. Adamczyk, Darryl G. Thelen

**Affiliations:** 1 Department of Biomedical Engineering, University of Wisconsin-Madison, Madison, WI 53706, USA; 2 Department of Mechanical Engineering, University of Wisconsin-Madison, Madison, WI 53706, USApeter.adamczyk@wisc.edu (P.G.A.)

**Keywords:** shear wave tensiometry, field-based measurement, inertial measurement units, locomotion, muscle–tendon mechanics, Achilles, noninvasive, work loop

## Abstract

Muscle–tendon power output is commonly assessed in the laboratory through the work loop, a paired analysis of muscle force and length during a cyclic task. Work-loop analysis of muscle–tendon function in out-of-lab conditions has been elusive due to methodological limitations. In this work, we combined kinetic and kinematic measures from shear wave tensiometry and inertial measurement units, respectively, to establish a wearable system for estimating work and power output from the soleus and gastrocnemius muscles during outdoor locomotion. Across 11 healthy young adults, we amassed 4777 strides of walking on slopes from −10° to +10°. Results showed that soleus work scales with incline, while gastrocnemius work is relatively insensitive to incline. These findings agree with previous results from laboratory-based studies while expanding technological capabilities by enabling wearable analysis of muscle–tendon kinetics. Applying this system in additional settings and activities could improve biomechanical knowledge and evaluation of protocols in scenarios such as rehabilitation, device design, athletics, and military training.

## 1. Introduction

The study of biomechanics is often conducted with the purpose of understanding human movement as it pertains to everyday life. In pursuit of this knowledge, it is of value to study individuals in an array of activities that reflect their daily tasks or athletic engagements outside of the laboratory environment. While certain tasks such as treadmill walking or jumping may be readily tested and analyzed in a motion capture space, others are more difficult to replicate [1]. Many studies have labored to mimic locomotor tasks, including ascent and descent on sloped surfaces and stairs, through creative and complex laboratory setups [2,3,4,5,6,7]. The alternative to bringing these activities to the laboratory equipment is to translate the equipment to the natural environment where these activities typically occur.

Wearable technologies have attempted to enable the portable capture of biomechanical measurements. Traditional lab-based assessments of muscle–tendon forces are indirect. Motion capture measures movement kinematics, while force plates record external forces [8,9,10]. Complex musculoskeletal models then estimate the underlying internal forces that produced the observed movement [11,12,13,14,15,16]. With the emergence of wearables, kinematics can now be easily collected via inertial measurement units (IMUs) and smartphone-based video motion analysis software [17,18]. Foot–floor kinetics can be tracked by pressure-measuring insoles [19,20,21]. Recently, we introduced wearable shear wave tensiometry that enables the measurement of tendon loads during dynamic activity [22]. Individually, these technologies have provided valuable types of information regarding unconstrained human movement. For example, wearable kinematic sensors yield clinically relevant values such as passive or active range of motion, and tensiometry can be used to measure muscle loading and assess muscular contributions to joint mechanics [22].

The fusion of wearable kinematic and tensiometry measures could enable muscle power and work analyses during real-world movements such as manual labor, sports, physical training, and rehabilitation [23,24,25,26,27,28,29,30,31,32,33,34,35,36]. Traditionally, laboratory measures of joint kinematics and kinetics have been coupled to evaluate joint power output and positive and negative work production [2,37,38,39,40,41]. Prior wearable studies have used insoles with IMUs to estimate joint kinetics [21,42,43,44,45,46,47,48,49,50], which would enable joint energetic analysis outside the laboratory. Musculoskeletal modeling efforts can be used to extend these measurements to estimate power and work production at the muscle–tendon level [42,46,47,51,52,53,54,55], but require an extensive set of assumptions to resolve muscle redundancy [56,57,58,59,60]. As a wearable technology, tensiometry avoids this problem by directly assessing tendon tissue loads. This circumvents much of the musculoskeletal modeling step and many of its assumptions.

This study aimed to enable field-based estimates of muscle-specific work and power using only wearable sensors. To do this, we fused triceps surae muscle–tendon load assessed via tendon tensiometry with ankle and knee kinematics estimated from inertial pose sensors. Using this system, we investigated stride-by-stride triceps surae work production during outdoor walking on a course with varying slope. Previous laboratory studies have found that with inclining slope, positive ankle power, and work increase, negative power and work decrease in magnitude, and net ankle work increases in response to incline angle [2,3,37,39]. We expected to observe similar trends, in that net soleus work and net gastrocnemius work would both correlate positively with ground incline. Furthermore, we aimed to explore these trends across a greater range of natural inclines than we previously studied, with the hypotheses that steeper positive (inclining) slopes would further increase peak muscle force and total work, and steeper negative slopes would increase triceps surae muscle force at midstance and reduce total work [22]. This study is unique in its aim to generate muscle-specific estimates of work through the use of tensiometry. By establishing the ability to capture and combine both kinematic and kinetic data and estimate muscle–tendon work and power, this wearable muscle power measurement system could enable exciting new investigations of locomotion energetics beyond the traditional laboratory setting.

## 2. Materials and Methods

Twelve healthy young adults participated in this study after giving their written informed consent (Table A1). Participants were deemed eligible if they self-reported the ability to perform typical activities of daily living without ambulatory aids or experiencing adverse symptoms; were not under treatment for any infectious diseases; were free from any psychiatric, musculoskeletal, cardiovascular, or neurological conditions, and had no serious leg injury or surgery within the past year. The study was conducted in accordance with the Declaration of Helsinki, and the protocol was approved by the University of Wisconsin-Madison Institutional Review Board (protocol 2018-0487, approved 30 August 2020). Data from one participant were omitted because of poor data quality, likely due to inadequate contact between the tensiometer and tendon. Thus, eleven participants’ data were included in the analysis (5 F/6 M, mean ± standard deviation: 23.3 ± 2.5 years, height 1.77 ± 0.13 m, body mass 76.5 ± 13.6 kg, BMI 24.1 ± 2.2 kg/m^2^).

**Figure 1:**
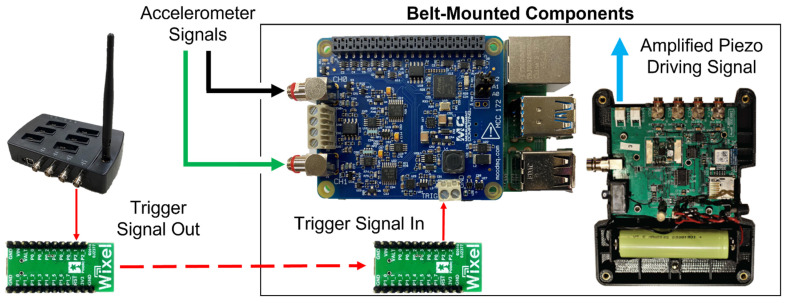
Synchronization signals were delivered wirelessly from the XSens MVN Awinda Station (**left**) via Pololu Wixels to the tensiometer’s MCC 172 DAQ HAT trigger pin (**center**) [63,64,65]. Participants wore a belt to accommodate these components for driving the tapper and recording the accelerometer data.

The tendon tensiometer unit consisted of a piezoelectric actuator stack (PK4JQP2, Thorlabs, Newton, NJ, USA) embedded in a tendon-tapping mechanism and two miniature accelerometers (Model 352C23, PCB Piezotronics, Depew, NY, USA) embedded in silicone, as previously described [22]. The actuator introduced microscopic shear waves by applying impulsive “taps” to the tendon through the skin, which were recorded by the accelerometers further along the tendon to compute shear wave speed [22,61,62]. To ensure data quality, tensiometers were mounted over both Achilles tendons on each participant using reusable neoprene straps. Tensiometers were placed over the free tendon region, with the distal accelerometer approximately level with the medial and lateral malleoli based on palpation and visual inspection, and allowing for clearance of footwear during maximum plantarflexion. Upon data inspection after the collection period, the side with the higher peak cross-correlation coefficient between the two accelerometers’ signals was used for analysis (9L/2R). IMUs were secured to the feet, shanks, thighs, and sacrum (MTw Awinda, XSens Gmbh, Enschede, The Netherlands). On a waistbelt, participants wore the tensiometer electronics: a custom piezo driver and data logger (Figure 1) [22].

The driver excited the piezoelectric tendon tapper at 100 Hz (a 2 ms pulse every 10 ms), and the data logger was comprised of a Raspberry Pi 4B (Raspberry Pi Foundation, Cambridge, UK) and an IEPE Measurement HAT (Measurement Computing Corporation, Norton, MA, USA), which recorded accelerometer data at the maximum nominal rate of 51.2 kHz (actual achieved rate of 50.66 kHz) per channel and logged it to a disk through a Python program. For each trial, a trigger signal was sent from the MTw Awinda Station to the data logger to initiate synchronized collection, via a pair of general-purpose programmable wireless modules running a remote pin-mirroring program (Pololu Corporation, Las Vegas, NV, USA) (Figure 1).

Following the consent process and donning the wearable system, participants completed a calibration task. Participants stood upright with the instrumented leg on a balance plate (BP5046, Bertec Corp, Columbus, OH, USA) [62] and their ankle axis, defined by the lateral and medial malleoli, aligned with the mediolateral axis of the plate. Participants were asked to perform voluntary anterior–posterior swaying while the vertical ground reaction force and center of pressure were monitored at 1000 Hz. To synchronize the balance plate data with the tendon tensiometry data, the plate was manually struck to induce a spike in both the vertical ground reaction force and accelerometer signals. After cross-correlating these signals to align the spike, the center of pressure and vertical force were used to calculate the net ankle moment. We then performed a linear regression between squared wave speed and ankle moment, as suggested by a tensioned beam model [61]. The participant-specific calibration was then used to convert wave speed measures to ankle moment in all subsequent tasks. Ankle moment was further converted to tendon tension in a later stage of processing (Table A2). The mean fit between moment *M* (units Nm) and wave speed *S* (units m/s) was:

*M =* 0.05(*S*^2^) − 17.0, (1)

where 0.05 represents the moment per unit of squared wave speed, *S*^2^, and 17.0 represents the unloaded tendon wave speed. Individual participants’ calibration curves fit their data with moderately strong correlations (R^2^ = 0.85 ± 0.08) (Table A2).

**Figure 2:**
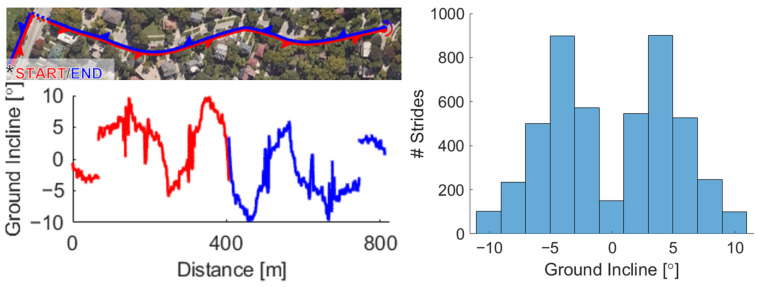
The outdoor course covered a continuous range of inclines from −10° to +10°. Note that a crosswalk was omitted for safety (shown as a gap in the trace). This path was traversed in both directions to balance inclines and declines, yielding 4777 strides for analysis across 11 participants. Grouping strides by two-degree bins of slope, at least 100 strides were included in each bin.

Before heading outside, participants completed a 30-s treadmill walking trial at a self-selected speed (mean 1.2 ± 0.16 m/s). They then traversed an 800-m outdoor course, which consisted of a range of slopes between −10° and +10° (Figure 2); this was a 400-m hill along neighborhood sidewalks that was navigated in an out-and-back path, so that a symmetrical spread of slopes was acquired. The treadmill walking and sway trials were repeated at the conclusion of the testing period to verify the calibration.

### 2.1. Data Processing

Accelerometer signals were band-pass filtered using a fourth order Butterworth filter with lower and upper cutoff frequencies of 150 and 1500 Hz, respectively. Then tap events were identified across the entire trial. This was done by cross-correlating a representative tap signal with the first accelerometer signal and then finding the times of peak correlation, which corresponded with a tap event. Using the first 4 ms of data following each tap event, cross-correlation of the first and second accelerometer was used to determine the wave travel time between accelerometers [22,61]. Wave speed was then computed by dividing the axial spacing between the two accelerometers (8 mm) by the travel time. The wave speed time-series data were split into strides at an approximate heel-strike event defined by a peak in accelerometer data filtered to 10–50 Hz [66,67].

For each stride, a slope value was assigned by mapping participant position onto a topographical map of course incline [22]. This map was created by collecting positional data from an IMU suit worn by a person rolling a bicycle with an additional IMU affixed to its crossbar to measure slope. For both the mapping sessions as well as all participant trials, displacement along the course was defined as the cumulative horizontal displacement of the pelvis unit between subsequent time points. The final topographical map was achieved by scaling these positional data to the known distance of each section of the course, averaging two measurements of the incline at each point (from outward and returning passes), and applying a moving 2-m median filter to the incline data (Figure 2).

Inspection of the data revealed a slight delay between the start times of the data collected from the IMU and tendon tensiometry systems. The delay was ultimately traced to non-deterministic timing in the wireless module used to transmit the synchronization trigger (Wixel, Pololu; delay up to 100 ms) [64]. To retroactively correct for this, cross-correlation was used to align ankle flexion angle with wave speed during the first 10% of the gait cycle from level strides; this method was devised based on observed alignment in a previous study and tested on the relevant data set [68], reproducing synchronous timing within 0 ± 1% gait cycle. The mean offset was then used to align the beginning of each trial, and all strides were split using the accelerometry-defined heel strikes.

### 2.2. Estimation of Outcome Metrics

Spatiotemporal metrics were determined from IMU measures and used in part as criteria for omitting certain strides due to errors in the proprietary IMU motion reconstruction (MVN Analyze, XSens Gmbh). Stride times were defined by the total duration of each stride according to the IMU timestamps between successive heel strikes. Likewise, stride length was calculated as the total horizontal displacement between heel strikes. Walking speed was then computed as the quotient of stride length and time. If the recorded stride length was greater than 3 m, the stride was omitted. Finally, we computed work against gravity for each step knowing the elevation gain, which was determined by step length and the slope of the terrain. Work against gravity per step was calculated as 50% of the product of body weight with the length of each stride and the tangent of its incline.

To estimate the power and work of each unit of the triceps surae, we combined tendon load estimates from tensiometry with muscle–tendon velocity estimates obtained from IMU movement data. Because muscle and tendon interact in series and change length nonuniformly throughout movement, muscle vs. tendon length can only be differentiated via imaging such as ultrasound [69,70]. Long duration ultrasound measurements are not viable, so we estimated only the combined length, power, and work of each muscle–tendon unit (MTU) as a whole. We assumed that force in the tendon is equal to force in the muscles due to their series arrangement. We combined this force with muscle–tendon velocity estimates to obtain muscle–tendon power, which was integrated to estimate work. Estimates of muscle–tendon velocity were obtained from joint angle measurements and moment arms of the Achilles and proximal gastrocnemius tendons about the ankle and knee, respectively [56,71,72]. To prepare lookup tables for the determination of time-series moment arm data, generic relationships of joint moment arms, $R_{ankle}$ and $R_{knee}$ (units m), to ankle dorsiflexion, θ, and knee flexion, Φ (units degrees), were identified [56,73]:

(2) $$R_{ankle}\left(\theta \right)=1.4*10^{-6}\;\;\theta^{2}\;–1.7*10^{-4}\;\theta +4.4*10^{-2},$$

(3) $$R_{knee}\left(\Phi \right)=-2.0*10^{-6}\;\Phi^{2}+3.8*10^{-4}\;\Phi +1.8*10^{-2},$$

where coefficients represent quadratic best fits to previous data and a neutral pose of straight-legged upright standing defines zero angles [56,73]. From these relationships, moment arms were determined for every time point. To estimate muscle–tendon velocity due to rotation about the ankle and knee, ankle and knee moment arms were then multiplied by the instantaneous angular velocity of their respective joints. As the gastrocnemius is a biarticular muscle, gastrocnemius velocity, $V_{G}$ (units m/s), was estimated by summing the velocity contributions from the ankle and knee rotations, while soleus velocity, $V_{S}$ (units m/s), was estimated using only the velocity due to rotation about the ankle [56,73,74]:

(4) $$V_{S}=R_{\mathrm{ankle}}\left(\theta \right)*\dot{\theta }\;$$

(5) $$V_{G}=R_{\mathrm{ankle}}\left(\theta \right)*\dot{\theta }-R_{knee}\left(\Phi \right)*\dot{\Phi }.$$

These muscle–tendon velocities were ultimately multiplied by the force in each MTU to estimate instantaneous MTU power generation.

We estimated the excursion of each MTU to investigate the different behaviors of the two muscles. Excursion of each muscle–tendon unit, defined as the difference between the current length and the length in the neutral pose, was computed by symbolically integrating Equations (2) and (3) with respect to measured knee and ankle angles.

The force in each MTU (soleus, gastrocnemius) was estimated from Achilles wave speed and a simple load distribution model. After converting wave speed values to ankle moment (Equation (1)), Achilles tendon force, *F* (units N), was determined by dividing moment, *M* (units Nm) (Equation (1)), by instantaneous ankle moment arm, $R_{ankle}$ (units m), computed from instantaneous joint angles using Equation (2):

(6) $$F=\frac{M}{R_{ankle}}.$$

This total Achilles force is distributed across the two muscle–tendon units that load the Achilles: the gastrocnemius and soleus. Data-derived apportionment of Achilles force to the individual units would depend on individual-specific and time-varying motor control, muscle mechanics, and posture; accounting for these factors would require adding electromyography, imaging, and modeling. To avoid this complexity and retain a mobile, wearable system for this study, we assumed a constant proportion of load for each muscle–tendon unit based on the physiological cross-sectional area of the muscle [75]. A total of 65% of the force was attributed to the soleus, and the remaining 35% was attributed to the gastrocnemius. Soleus force, $F_{S}$, and gastrocnemius force, $F_{G}$ (units N/kg), were both normalized to participant body mass, *m* (units kg):

(7) $$F_{S}=0.65F/m,$$

(8) $$F_{G}=0.35F/m.$$

Instantaneous muscle–tendon powers generated by the soleus, $P_{S}$, and gastrocnemius, $P_{G}$ (units W/kg), were defined as the negative product of their respective muscle force and lengthening velocity data for each time sample:

(9) $$P_{S}=-V_{S}*F_{S}\;$$

(10) $$P_{G}=-V_{G}*F_{G}.$$

Numerical integration of muscle power data yielded net work of the soleus, $W_{S}$, and gastrocnemius, $W_{G}$ (units J/kg), over each stride:

(11) $$W_{S}=\int P_{S}dt\;$$

(12) $$W_{G}=\int P_{G}dt.$$

Similarly, positive and negative work were obtained by numerical integration of positive and negative power, respectively. We also plotted normalized muscle force vs. muscle–tendon excursion for both muscles to visualize the work loop plots of the triceps surae.

### 2.3. Statistical Analyses

For each participant, we first normalized the Achilles tendon wave speeds over a gait cycle to participant-specific mean peak wave speed in the level condition. We then averaged the normalized wave speed patterns across participants at different slopes, from −10° to +10° in 2° bins, to produce mean curves for plotting. For each step of each participant, we determined the net, positive, and negative muscle–tendon work and the slope of the terrain. We then subtracted participant-specific mean net work in the level condition to obtain the change in net work from the level condition, accounting for inter-participant differences. Separately for inclines and declines, we fit a line to estimate the sensitivity of the change in net work to the slope angle of each step, for both the soleus and gastrocnemius. Linear regression coefficients were evaluated at the 0.05 statistical significance level.

## 3. Results

Participants navigated the course in 10.3 ± 0.8 min (mean ± standard deviation) with a speed of 1.31 ± 0.10 m/s and a stride length of 1.60 ± 0.25 m in the level condition (mean ± standard deviation). On average, 63 strides per participant were excluded due to data quality concerns, arising from poor sensor contact and temporary hardware failures. A total of 4777 strides were included for analysis, with a mean of 434 strides per participant.

**Figure 3:**
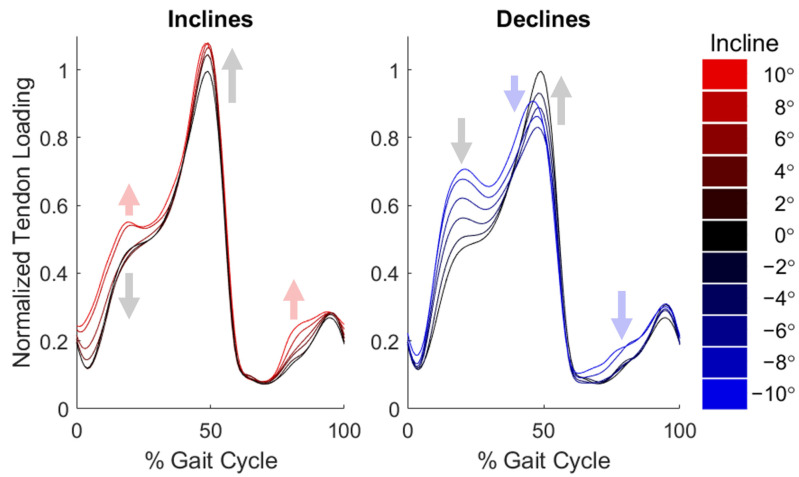
Mean normalized wave speed trajectories over the gait cycle in each slope bin across 11 participants. Gray arrows indicate trends with increasing slope across moderate slopes, while colored arrows indicate trends unique to steep slopes.

Achilles tendon wave speed measures were sensitive to changes in slope [22] (Figure 3). Across slopes up to +6°, wave speed at midstance (around 20% of the stride) decreased with slope. At the steepest inclines (greater than 6°), midstance wave speed increased. Across slopes greater than −6°, peak wave speed increased with slope. At the steepest declines (below −6°), peak wave speed increased compared to peak wave speed at 6° (blue arrow). Finally, additional regions of difference were observed in the steep conditions: steep incline wave speeds were increased both in early stance (0–20% gait cycle) and in mid-swing (70–90% gait cycle), and steep decline wave speeds were increased throughout early and mid-swing (60–90% gait cycle).

**Figure 4:**
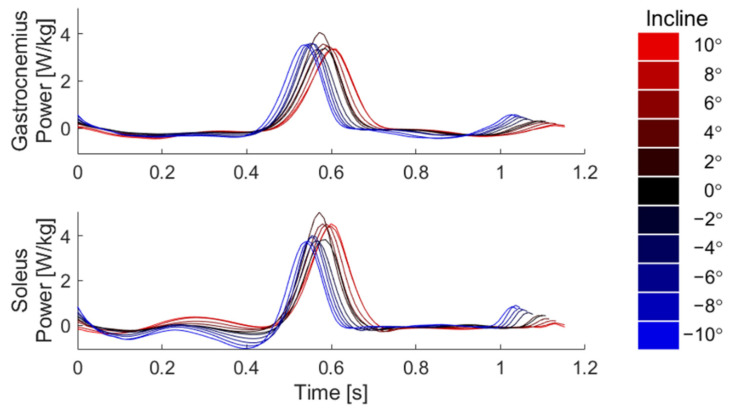
Muscle power derived illustrate slope-dependent differences in timing and magnitude of positive and negative work.

**Figure A1:**
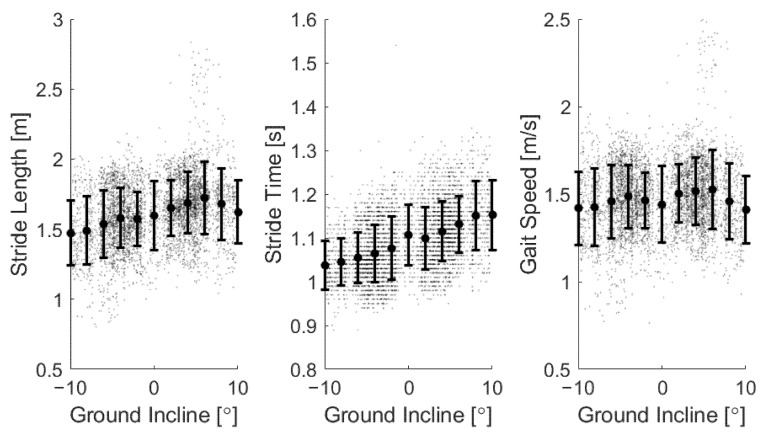
Spatiotemporal metrics for each stride, with mean and standard deviation of each slope bin.

Both the gastrocnemius and soleus displayed diminished swing-phase power as well as increasing stride time with increasing slope (Figure 4). Soleus power in midstance (0.25–0.45 s) was generally negative in decline conditions and positive in incline conditions. While peak gastrocnemius power was relatively uniform across slopes, peak soleus power generally increased with slope. Finally, while absolute stride time increased with slope, key features such as peak power and peak swing power occurred at the same relative percentage of the gait cycle. As stride length also tended to increase with slope, walking speed was thus relatively constant across slopes (Figure A1).

**Figure 5:**
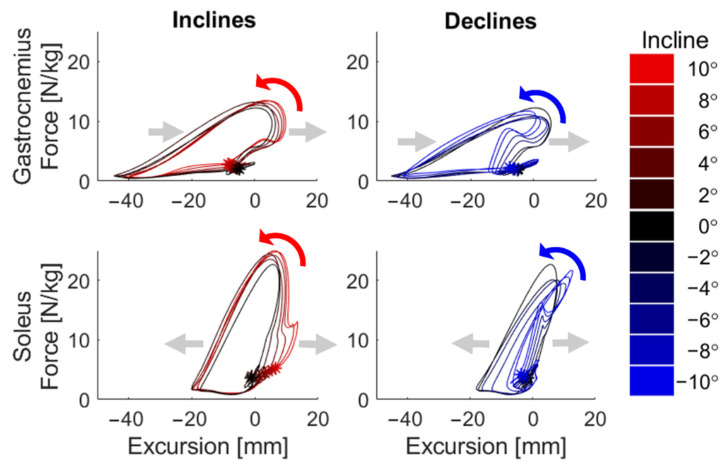
Muscle–tendon work loops, i.e., plots of force vs. excursion of the muscle–tendon length; work is the area within the loop, positive if the loop progresses counter-clockwise. The work loops illustrate increasing work production with increasing incline angle. Gray arrows indicate trends with increasing slope across all slopes, while colored arrows indicate loop progression direction. All loops begin at heel strike, designated by an asterisk, and follow a counter-clockwise direction. Length is presented as “excursion”, representing the change in muscle–tendon length relative to its length in an upright posture.

**Figure A2:**
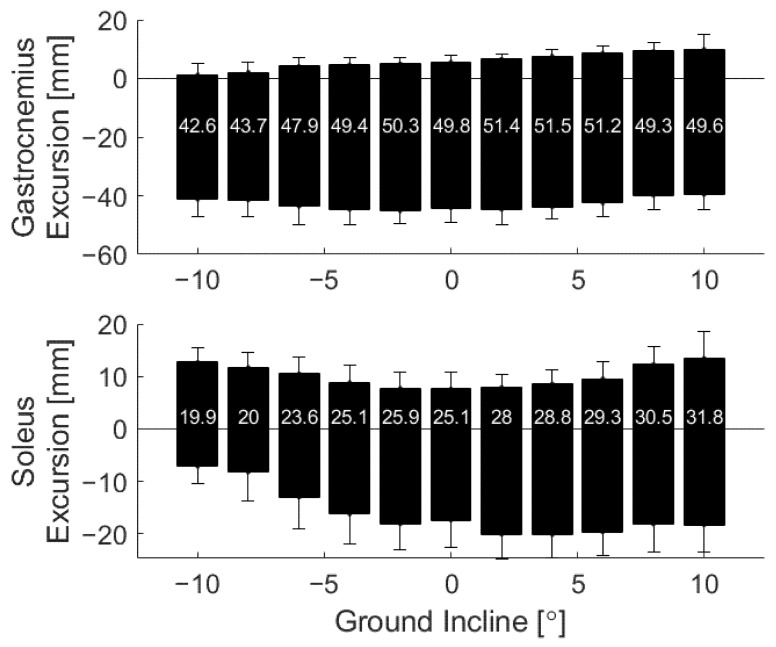
Mean excursion of the soleus and gastrocnemius muscle–tendon length, relative to the length in an upright posture, at each of the slope bins. Error bars represent one standard deviation.

**Figure A3:**
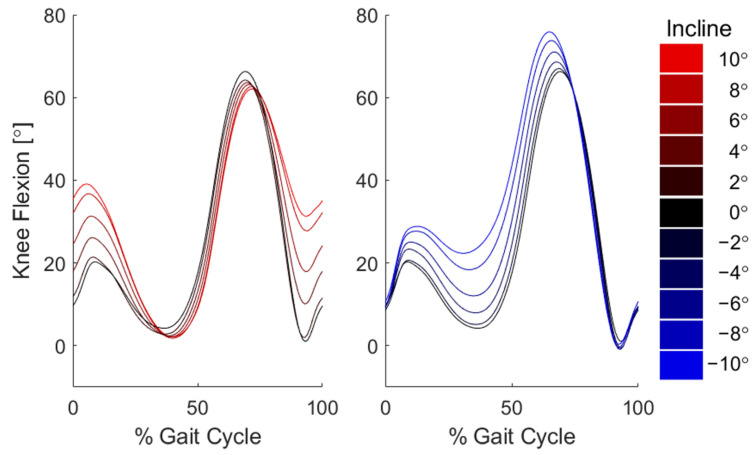
Mean knee flexion angles from each slope bin.

**Figure A4:**
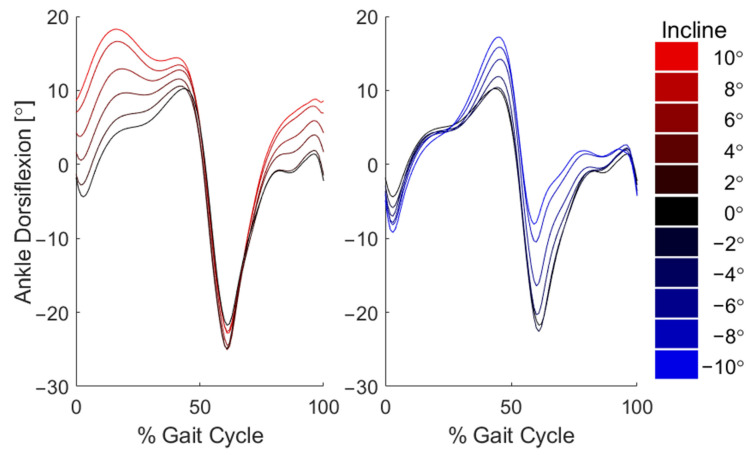
Mean ankle flexion angles from each slope bin.

Work loop plots exhibited systematic variations with ground incline (Figure 5). All slopes induced positive net work production out of the triceps surae, despite the fact that net work done against gravity was negative on downhill slopes (Figure 5). Gastrocnemius work loops displayed excursion ranges that shifted with slope (Figure 5, also see Figure A2), reflected in phases of increased knee flexion (see Figure A3). Conversely, soleus work loops were observed to expand in excursion with slope (Figure 5), reflecting the widening of ankle flexion ranges (Figure A4).

**Figure 6:**
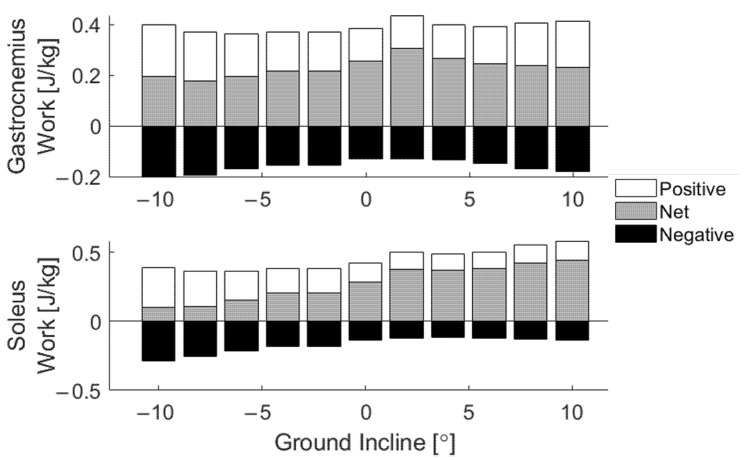
Zero-referenced positive, negative, and net work from each slope bin.

To quantify the effect of slope on individual muscle output, integration of power over time yielded work estimates (Figure 6). Soleus net work increased with ground incline, marked by an increased magnitude of negative work on increasingly negative slopes and increased positive work on increasingly positive slopes. Gastrocnemius net work varied nonlinearly with ground incline, marked by an increased magnitude of negative work with increasing absolute slope (both decline and incline). Overall, both muscles maintained positive mean net work at all slopes.

**Figure 7:**
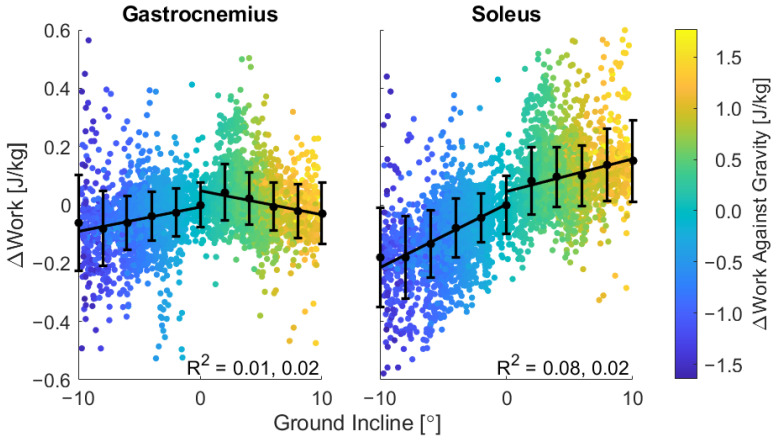
For each of 4777 strides from 11 participants, change in net work relative to participant-specific mean net work in the level walking strides. Linear regressions performed separately for negative and positive slopes illustrate differing trends in gastrocnemius vs. soleus muscle function.

**Figure A5:**
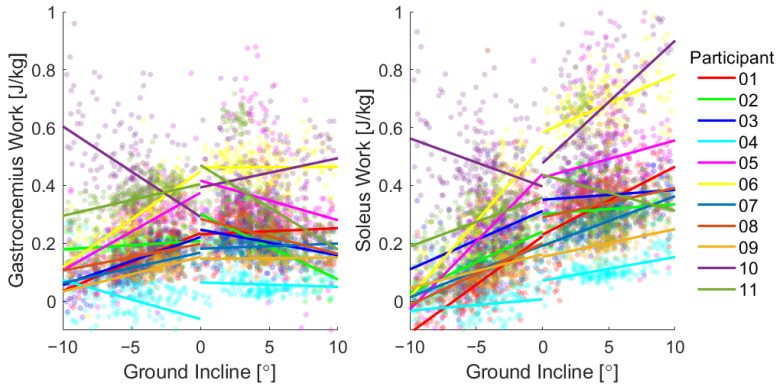
Estimated net gastrocnemius work was less sensitive to incline, whereas net soleus work for each participant generally increased with ground incline (observe positive slopes for all but two lines).

Relative to participant-specific mean net work in the level condition change in net work scaled with slope (Figure 7). Inter-participant differences accounted for much of the variability, demonstrating a few unique strategies participants used to navigate this range of slopes (Figure A5). On slopes from −10 to 0 degrees, soleus net work increased with slope by 0.02 J/kg per degree; on positive slopes, soleus net work increased with slope by 0.01 J/kg per degree. Gastrocnemius net work was less sensitive to slope, increasing with slope by 0.008 J/kg per degree on slopes from −10 to 0 degrees, but decreasing with incline by 0.008 J/kg per degree on positive slopes. Work against gravity ranged from −1.5 J/kg in decline strides to +1.5 J/kg in incline strides, increasing slightly with muscle work within a given slope bin (Figure 7).

## 4. Discussion

This study introduces a wearable system of IMUs and tensiometry to characterize triceps surae kinematics, kinetics, and work production during sloped walking outdoors. Tensiometry tracks tendon loading based on the wave speed propagating in the tissue. The wave speeds and tendon forces measured in this study reflect trends previously demonstrated on mild slopes [22]. At steeper slopes (beyond ±6°), we did observe an apparent difference in strategy. At steep inclines, Achilles tendon loading through early and midstance increased as opposed to the decrease observed in mild slopes. In a similar reversal of trends, peak loading in terminal stance increased at steep declines, in contrast to the decrease noted in mild declines. These non-monotonic trends are not reflected in the corresponding ankle or knee angles, which display linear increases or decreases in flexion at each phase of the gait cycle. This discrepancy of trends indicates the value of obtaining measures of both kinetics and kinematics in characterizing muscle–tendon function.

Tensiometry provides for a continual assessment of muscle–tendon kinetics, including during periods without ground contact. We have previously observed elevated wave speeds during the late swing phase in level walking, corresponding with passive triceps surae stretch due to dorsiflexion motion forced by the antagonist muscles (e.g., tibialis anterior) [22,56,61,68]. In this study, we found that swing-phase tendon loading was relatively insensitive to mild slopes, despite marked changes in both ankle and knee flexion (e.g., compare Figure 3 to Figure A3 and Figure A4). However, the steepest slopes of both inclines and declines induced loading earlier in the swing phase, likely due to the use of a tripping avoidance behavior needed to ensure ground clearance. Indeed, the observed trends of knee and ankle flexion on slopes are consistent with those previously reported in laboratory studies [3,76].

The fusion of IMU and tensiometry data enabled an analysis of the variation of gastrocnemius and soleus excursion and work production with slope. Our work loop plots qualitatively agree with previous laboratory work [56], though we did estimate greater net work than observed during level walking with lab-based tendon tensiometry. This is likely due to the difference in methodology: the in-lab study used motion capture to track joint angles, which may differ slightly from IMU-derived measurements [17]. In incline or decline conditions, work loops deviate in specific ways from the level condition. An increased soleus excursion during early stance (0–20%) on inclines coincides with an impact impulse load on the muscle (Figure 5). This results in increased muscle–tendon length through midstance, which allows for the increased midstance positive power stroke (20–40%), aiding to elevate and propel the body up the incline (Figure 4). Conversely, the early stance portion of soleus work loops from negative slopes shrink and invert to indicate negative work, while the soleus is resisting forward rotation of the shank to decelerate the body and control descent (Figure 5) [2,3,4,37,38,76]. At all slopes, soleus force increases by a similar magnitude during midstance; however, this occurs at distinct muscle–tendon lengths that scale with slope. This alone could explain the changed capacity and tendency of the soleus to perform positive work, perhaps stabilizing the system in preparation for the power generation during push-off. Considering the gastrocnemius, excursions due to ankle flexion are largely mitigated by increases in knee flexion, resulting in a relatively uniform range of excursion that shifts with slope (Figure 5 and Figure A2). Knee flexion leaves the gastrocnemius in an already shortened state and unable to exert full power at the ankle, flattening the power curve in stance (Figure 5). Some of these trends are similar to previous laboratory values of ankle power [3], but the ability to estimate muscle–tendon behaviors allows for an expanded view of these nuanced differences between the muscles.

Our reported trends in work agree with previous studies of joint-level work. In particular, the steady increase in net soleus work clearly reflects how ankle work scales with ground incline (Figure 6) [2,37]. This is marked by an increased magnitude of negative work in decline conditions and increased positive work in incline conditions (Figure 6). As stated above, these changes can be attributed to increased braking in early and midstance when traversing declines and increased power generation throughout stance and push-off in walking up inclines. When translating the limited changes in gastrocnemius power to summary metrics of net work, work is predictably less sensitive to slope. Interestingly, the negative gastrocnemius work increases in magnitude with increasing absolute slope (both decline and incline), the same trend observed in increasing mean knee flexion (Figure 6 and Figure A3). In future work, tensiometry on the patellar tendon will aim to illuminate changes in knee extensor kinetics to illustrate the interaction between knee and ankle work. This additional information may elucidate complex mechanisms such as power transfer through strut-like muscle behavior.

Previous laboratory studies have reported shifts in net, positive, and negative work among the lower extremity joints depending on slope. Several studies in similar slope ranges of −10° to +10° depict increasing ankle work with incline, including increased positive work uphill and increased negative work downhill [3,37]. At steeper slopes from −20° to +20°, trends continue with increased positive ankle work on positive slopes and increased negative ankle work on negative slopes [2,4,39]. Interestingly, these studies reported a more pronounced shift in work when switching from level to 6° compared to 6° to 12° or 12° to 18° [2,4]; this could suggest switching strategies as the hip and knee contribute more work to raising the leg [3,39]. Magnitudes of work in these studies range up to twice those reported here, likely arising from methodological differences and the aggregation of joint work rather than muscle work [37,77].

In this study, magnitudes of soleus and gastrocnemius work were compared to the total work against gravity at each step. Despite experiencing an overall negative work against gravity in negative slopes, both soleus and gastrocnemius net work were positive at all slopes. This observation is consistent with a prior laboratory study which found gastrocnemius work to be positive at slopes ranging from −6° to +6° [76]. At the steepest upward slopes, soleus work output was approximately equal to one third of the work done against gravity. Within a slope bin, work against gravity subtly increased with muscle work (Figure 7), as seen in the slight change of color across the vertical spread of strides. Such variability across all slopes may be largely attributed to inter-participant variability (Figure A5); for example, certain participants may have increased their stride length more than others or may prefer to do more work with the knee and hip [3,39]. The remaining spread of data may be due to uncontrolled factors in the outdoor environment influencing step-by-step behavior, or by imperfections in the slope-mapping procedure. The relatively limited number of strides at certain slopes (−10°, 0°, +10°) may lead to greater uncertainty in those bins. However, at least 100 usable strides were collected in each bin, validating the inclusion of these bins for comparison with an extension of the trends from the mild slopes (Figure 2).

There were limitations within our methods that should be considered in interpreting the results. The wireless triggering method between the kinematic and kinetic measurement systems resulted in a delay varying between 10 and 100 ms for every trial. At the preferred cadence of the participants in this study, this equates to offsets of approximately 1–10% of the gait cycle. As power and work calculations are highly dependent on the time-sensitive relationship between force and movement, it was thus necessary to correct for these delays. After applying the correction described in the Methods section, a systematic sensitivity study of the effect of incremental shifts in the data on resultant calculations of work output demonstrated that even 1% gait cycle offsets in either direction could affect work estimates by approximately 20%. However, for up to at least 10% gait cycle offsets in either direction, the overall trends in the work output calculations remained unchanged within and across participants. Thus, while a hardwired synchronization will be preferred in future work, we are nevertheless confident in the applied strategy and its ability to yield reasonable absolute outputs with meaningful relative trends.

Relative to our previous experiment [22], data loss was reduced by improvements in hardware and procedure, though some issues remain to be addressed. For the one omitted participant, the problem was largely attributed to hardware malfunction throughout the trial, including poor cable connections and poor kinematic calibration; these issues likely arose due to shifting of the tensiometer upon embarking on the outdoor course, resulting in inadequate contact between the tensiometer and tendon. To a lesser extent, similar effects were noted in data from other participants, causing the occasional stride to be excluded. Finally, certain strides were also excluded due to poor signal quality likely caused by imperfect sensor contact with the skin directly over the Achilles tendon. However, these challenges have improved from past studies through the evolution of the device design. As these implementations continue to improve, so too will the data quality.

A final source of potential error lies in the limitations of the data acquisition hardware and processing methods. First, the accelerometers themselves have some inherent noise, in this case approximately 0.0001% to 0.0032% of the signal amplitude (greater for the second than for the first accelerometer due to attenuation as the shear wave propagates) [78]. Additional uncertainty appears in computing wave speeds, though cosine fitting of the cross-correlation permits subsample resolution in determining optimal signal delay; this procedure results in theoretical errors less than 1% of the sample period [22,79]. Then, the dynamic IMU pitch angle measurements are reported to be accurate within 0.75° [63]. Next, the calculation of individual muscle forces and excursions in this study relies on literature estimates of physiological cross-sectional areas and tendon moment arms. We assumed a simple static assumption of force allotment between the soleus and gastrocnemius muscles based on the physiological cross-sectional area [75], which clearly ignores potential variation in motor control and muscle mechanics over time and posture and across participants. Differing activation levels could be accounted for via simultaneous tracking of muscle EMG, and force–length and force–velocity relationships could be approximated through modeling. Next, while a characteristically similar population of healthy young adults was used to ascertain the generic relationships of ankle flexion angle to moment arms [56,73], a participant-specific approach using ultrasound may be preferred if absolute values of excursion, power, and work are of value. Finally, we only assessed the net muscle–tendon work and are not able to delineate energy exchange between the muscle and tendon [70]. Simultaneous ultrasound measures of muscle fascicle excursions would be needed to analyze dynamic muscle–tendon interactions [69,80].

Interpretations of the estimated quantities afforded by this wearable system may be applied in numerous contexts. Employing this methodology in healthy athletes and those recovering from an injury such as Achilles tendon rupture could enable the assessment of kinetic limb symmetry in field-based maneuvers; by capturing triceps surae work and moment through our instrumentation, this work could expand and inform the types of tests necessary for a safe return to sport [81]. Toward preventative measures, this system could be applied to military training to quantify cumulative workloads and determine injury thresholds. Again, such findings would advise training guidelines in order to mitigate the high rates of overuse injuries incurred in these environments [82]. Similarly, this approach could be used to inform protocols for the prevention of lower-limb muscle and tendon injuries using accessible tasks in other fields such as athletics and ergonomics. Finally, this technology could be utilized to characterize the performance of devices such as orthoses and exoskeletons; measures of muscle–tendon function could supplement metabolic information in order to measure and regulate specific assistance in the local musculature. In much the same way, this wearable system could be employed to enhance biomechanical knowledge in many fields.

## 5. Conclusions

This work fuses wearable Achilles tendon tensiometry and lower-limb kinematic measures from IMUs to simultaneously assess muscle–tendon kinematics, loads, and work production during outdoor locomotion. By detecting different trends in gastrocnemius and soleus work with changes in ground incline, the combined use of tensiometry and IMUs demonstrates its potential to illuminate muscle-specific behaviors in unconstrained settings. The wearable muscle–tendon power analysis system could enable exciting new applications beyond the traditional laboratory setting, including rehabilitation, sports medicine, and athletic training.

## Data Availability

The data that support the findings of this study are available from the corresponding author upon reasonable request.

## Appendix A

**Table A1:** Participant characteristics for the analyzed healthy young adults.

Participant Number	Sex	Age (Years)	Height (Meters)	Weight (kg)	Body Mass Index (kg/m^2^)
1	M	23	1.87	79.4	22.8
2	F	20	1.65	68.0	25.0
3	F	25	1.83	83.9	25.1
4	M	28	1.78	86.2	27.3
5	M	25	1.87	88.5	25.4
6	M	24	1.93	86.2	23.1
7	F	19	1.70	63.5	21.9
8	F	24	1.68	63.5	22.6
9	F	24	1.52	47.6	20.5
10	M	22	1.92	86.2	23.4
11	M	22	1.78	87.0	27.8
Mean ± Standard Deviation	5 F/6 M	23.3 ± 2.5	1.77 ± 0.13	76.5 ± 13.6	24.1 ± 2.2

**Table A2:** Individual calibration parameters.

Participant Number	Regression Coefficient	Intercept	R-Squared
1	0.093	−28.51	0.87
2	0.044	−18.49	0.92
3	0.049	−10.15	0.71
4	0.030	−7.51	0.72
5	0.080	−45.14	0.91
6	0.033	−6.32	0.84
7	0.023	−6.55	0.94
8	0.037	−9.12	0.90
9	0.022	−2.17	0.83
10	0.074	−35.76	0.87
11	0.086	−51.65	0.74
Mean ± Standard Deviation	0.048 ± 0.03	−16.97 ± 17.36	0.85 ± 0.08
